# A new homogeneity index based on statistical analysis of the dose–volume histogram

**DOI:** 10.1120/jacmp.v8i2.2390

**Published:** 2007-03-20

**Authors:** Myonggeun Yoon, Sung Yong Park, Dongho Shin, Se Byeong Lee, Hong Ryull Pyo, Dae Yong Kim, Kwan Ho Cho

**Affiliations:** ^1^ Research Institute and Hospital National Cancer Center Goyang Korea

**Keywords:** homogeneity index, equivalent uniform dose, dose–volume histogram, intensity‐modulated radiotherapy

## Abstract

The goal of the present study was to develop a new dose–volume histogram (DVH)– based homogeneity index for effectively evaluating the dose homogeneity of intensity‐modulated radiotherapy plans. The new index, called the sigma‐index (“*S*‐index”) is defined as the standard deviation of the normalized differential DVH curve. In a study of 16 patients with brain tumors at our institution, the *S*‐index was found to vary from 0.80 to 3.15. Our results showed that the *S*‐index provides a more reliable and accurate measure of dose homogeneity than that given by conventional methods. A guideline for evaluating the dose homogeneity of treatment plans based on the *S*‐index and its relation to equivalent uniform dose is discussed.

PACS numbers: 87.53.Xd, 87.53.Tf

## I. INTRODUCTION

The radiotherapy objective of delivering a therapeutic dose to a well‐defined target while minimizing the dose to the surrounding normal tissue and critical organs requires optimization of

- conformity of the prescription dose to the planning target volume (PTV),
- dose homogeneity within the PTV, and
- dose to the surrounding normal tissue and critical organs.^(^ ^1^ ^–^ ^5^ ^)^

Accomplishing these goals requires a simple and universal scoring system for evaluating (and comparing) the dose distributions of various treatment plans. Expressing the dose distribution in the form of isodose curves or surfaces is useful, because the curves or surfaces graphically show the anatomic locations of regions of uniform dose, high dose, and low dose.

Like many physical quantities, dose is a scalar field, where “field” refers both to the region and to the value of the physical quantity in the region. Other types of quantitative evaluation of dose distribution are based on the dose–volume histogram (DVH) concept in either differential or integral form. Entities like the DVH (which are not used in other areas of physics) have proven to be invaluable for summarizing a three‐dimensional dose distribution in a two‐dimensional graph. The differential DVH (*d*DVH) and the integral DVH (*i*DVH) are both very useful for assessing tumor volume coverage and the dose delivered either to healthy tissue surrounding the target or to specific structures in the vicinity of the target. The DVH is thus a powerful tool for summarizing and quantifying complex dose distributions.

One of the most important benefits of a DVH is that provides an accurate assessment of homogeneity in the PTV. The presence of cold spots in a dose distribution will negatively affect tumor control, and an accurate evaluation of homogeneity in the PTV is therefore essential to the efficacy of the treatment plan.^(6)^

The conventionally used homogeneity index (*H*‐index) is defined as the ratio of the maximum dose $\left(D_{\text{max}}\right)$ in the PTV to the prescription dose $\left(D_{p}\right)$,^(^ ^7^ ^‐^ ^9^ ^)^ with a value closer to 1 indicating better homogeneity. The *H*‐index generally varies from 1 to 1.5 in real‐world patient treatment plans. The index's simplicity has led to its being extensively used for quantifying dose homogeneity in tumor volumes. However, although calculating this index is easy, the value obtained sometimes fails to accurately represent the homogeneity of the treatment plan, and obtaining a complete understanding of the dose homogeneity of the entire target requires consideration of the entire DVH curve.

**Figure 1:**
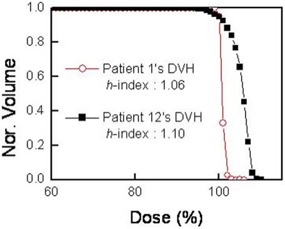
Apair of real patient dose‐volume histograms (DVHs) with conventional homogeneity indices (*H*‐indices) of 1.06 and 1.10. The normalized volume (Nor. Volume) in the y‐axis represents the fraction of the total volume *V*.

The *H*‐index also exhibits another problem. Fig. 1 shows a pair of real‐patient DVHs whose *H*‐indices are 1.06 (Patient 1) and 1.10 (Patient 12). The *H*‐indices indicate that the homogeneity of the plan for patient 1 is better than that for patient 12. However, the degree of this difference is unclear, because the index itself has no physical or clinical meaning, indicating the need for a new type of quantitative homogeneity index to be developed.

In the present study, we examined the dose homogeneity of treatment plans for brain tumors based on statistical analyses of *d*DVHs, and we developed a new index that provides objective scores of the dose homogeneity for the PTV. The intensity‐modulated radiotherapy (IMRT) plans for the brain tumors of 16 patients, as obtained using BrainSCAN version 5.2.1 software (BrainLAB, Heimstetten, Germany), were used to explore the effectiveness of the new homogeneity index. We also compared the new index with currently used homogeneity indices based on biologic effects on tumors.

## II. MATERIALS AND METHODS

### A. Currently used homogeneity indices

In addition to the *H*‐index, another homogeneity index called *HI* has been defined^(10)^ as

(1) $$HI=\frac{D_{2}-D_{98}}{D_{p}}\times 100\%\;,$$

where $D_{2}$ and $D_{98}$ represent the doses to 2% and 98% of the PTV, respectively. For example, $D_{98}$ indicates that at least 98% of the target volume receives this dose, and hence $D_{2}$ and $D_{98}$ are considered to be the maximum and minimum doses, respectively. Equation 1 shows that lower *HI* values indicate a more homogeneous target dose. Together with the *H*‐index, the effectiveness of the *HI* value in evaluating dose homogeneity is discussed and compared with our proposed new index later in this article.

### B. Statistical analysis of the DVH curve

**Figure 2:**
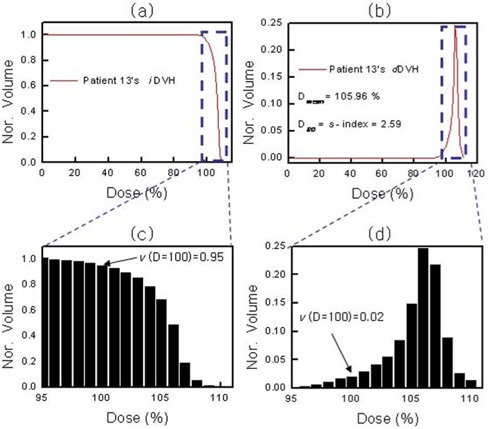
(a) Cumulative integral dose‐volume histogram (*i*DVH) for a patient. (b) Differential dose–volume histogram (*d*DVH) of the *i*DVH in panel a. (c) Magnification of the region marked in panel a. (d) Magnification of the region marked in panel b. Nor.=normalized volume.

The *i*DVH is a plot of the volume of a given structure that receives at least a certain dose, and the *d*DVH is a plot of the volume receiving a dose within a specified dose range. Because of its simplicity, the *i*DVH has been found to be more useful, and it is more commonly used than the *dDVH* is. However, the *d*DVH is also useful in that it can provide unique information regarding the extent of dose variation within a structure. The *i*DVH can be defined as

(2) $$d\text{DVH}=-\frac{\Delta }{\Delta D}(i\text{DVH})=-\frac{\Delta }{\Delta D}(\frac{\nu_{i}(D_{i})}{V})=-\frac{d}{dD}(\frac{\nu (D)}{V})\;,$$

where $v_{i}$ is the *i*th volume element receiving a dose of at least $\left(D_{i}\right)$, and *V* is the total volume. In this study, the x‐axis and the number of points (or resolution, Δ*D*) in the *d*DVH curve are, respectively, the percentage dose normalized by the prescription dose and 1 point for each percentage dose. Fig. 2(a,b) shows examples of the *i*DVH and *d*DVH for a real patient at our institution, and Fig. 2(c,d) shows magnified histograms in the region of the prescription dose. As indicated by the arrows in Fig. 2(c,d), the meaning of the volume element differs between the *i*DVH and *d*DVH. The V(D=100)=0.95 in *i*DVH indicates that 95% of the total volume receives at least the prescription dose, and the V(D=100)=0.02 in dDVH indicates that only 2% of the total volume receives exactly the prescription dose.

As can be seen in Fig. 2(d), the differential form of the real‐patient DVH for the PTV is similar to a conventional statistical distribution [*f*(*x*)] of an independent variable *x* (the dose). The mean value of a random variable *x* is also called its expectation value and is defined as

(3) $$x_{\text{mean}}=D_{\text{mean}}=\sum_{i}x_{i}f(x_{i})=\sum_{i}D_{i}\times \frac{\nu_{i}}{V}\;\;.$$

Based on the statistical analysis, we can use the standard deviation of the dDVH curve to quantify the spread or dispersion of the average dose within a structure—that is, the degree of inhomogeneity. This number—that is, the standard deviation—is called the sigma‐index (*“S*‐index”) and is defined as

(4) $$s-\text{index}=D_{\text{SD}}=\sqrt{\sum {\left(D_{i}-D_{\text{mean}}\right)}^{2}\times \frac{\nu_{i}}{V}}\;\;,$$

where $D_{\text{SD}}$ represents the standard deviation of the dose.

As an example, Fig. 2(b) shows the mean dose $\left(D_{\text{mean}}\right)$ and the corresponding S‐index. These values indicate that the PTV receives, on average, 105.96% of the prescription dose, with a variation of 2.59%. In other words, most of the PTV will receive from 103.37% to 108.55% of the prescription dose.

## III. RESULTS AND DISCUSSION

**Figure 3:**
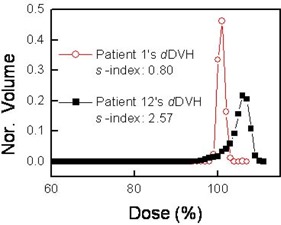
Sigma indices (*S*‐indices) for the two dose‐volume histograms (DVHs) shown in Fig. 1. Nor.=normalized volume.

To investigate how the new homogeneity index quantifies the dose homogeneity, we used the *S*‐index to reinvestigate the two DVHs shown in Fig. 1. The results are shown in Fig. 3. The *S*‐indices were 0.80 and 2.57 for the plans of patients 1 and 12 respectively. The difference of 0.04 in the *H*‐index between patients 1 and 12 does not clarify the distinction between the two DVHs in Fig. 3, but the difference in the *S*‐index clearly indicates that the homogeneity of the PTV dose is about three times better for patient 1 than for patient 12. In other words, the dose in the PTV ranges from 99.2% to 100.8% of the mean dose for patient 1, but from 97.43% to 102.57% for patient 12. These results show that the *S*‐index provides a better quantitative measure of the dose homogeneity than the *H*‐index does.

To further investigate how the conventionally used homogeneity indices (*H*‐index and *HI*) are related to the *S*‐index, we analyzed data from 16 patients at our institution. Table 1 indicates that a lower *S*‐index corresponds to a lower *HI*, as expected. However, some exceptions are seen, in that the *HI* can provide incorrect information on the dose homogeneity. Unlike the *HI*‐based system, the *H*‐index is not well matched with the *S*‐index, which suggests either that the *H*‐index should be avoided when attempting to quantify the degree of homogeneity accurately or that the definition of the *H*‐index should be modified.

**Figure 4:**
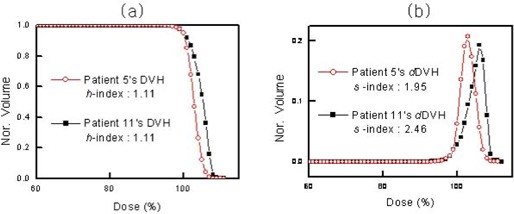
(a) Dose–volume histograms (DVHs) for two patients, with their corresponding conventional homogeneity indices (*H*‐indices). (b) Sigma‐indices (*S*‐indices) for the two DVHs in panel a. Nor.=normalized volume.

To illustrate the inadequate representation of dose homogeneity within the tumor volume by the currently used homogeneity indices, consider the two pairs of patient DVHs for brain tumors shown in Fig. 4. The two DVHs are normalized to facilitate comparison, where 95% of the PTV receives the prescription dose. Fig. 4(a) shows cases in which the DVH curves are quite different, but the *H*‐indices are identical (both H=1.11). The dose homogeneity of the DVH is clearly better for patient 5 than for patient 11. This mismatch between the real dose homogeneity in a PTV and its index is attributable to the *H*‐index being defined based on the point doses of the DVH rather than on the entire DVH curve. In contrast, Fig. 4(b) shows that the *S*‐indices for the two DVHs in Fig. 4(a) do indeed differ.

**Table 1:** Summary of various homogeneity indices sorted by the sigma index (S‐index)

Patient	S	*Indices* H	HI
1	0.80	1.06	2.8
2	1.05	1.05	3.8
3	1.56	1.06	5.9
4	1.68	1.09	6.2
5	1.95	1.11	8.0
6	2.00	1.16	9.1
7	2.03	1.09	8.4
8	2.18	1.11	10.0
9	2.33	1.09	9.1
10	2.37	1.10	10.1
11	2.46	1.11	9.5
12	2.57	1.10	10.7
13	2.59	1.10	10.5
14	2.77	1.12	11.7
15	3.03	1.13	12.4
16	3.15	1.13	13.2

**Figure 5:**
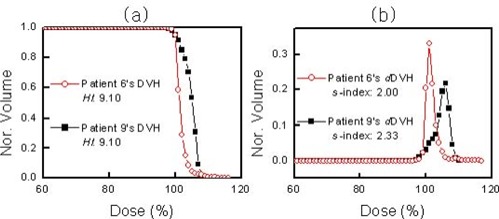
(a) Two dose–volume histograms (DVHs), with homogeneity index *HI*. (b) Sigma‐indices (*S*‐indices) corresponding to panel a. Nor.=normalized volume.

A comparison between the *HI* system and the *S*‐index reveals similar problems. Fig. 5(a) shows that the *HI* values for the DVHs of patients 6 and 9 do not accurately represent the dose homogeneity. The *HI* system indicates that the homogeneity of the two DVHs is the same, which contradicts the better homogeneity of the DVH of patient 6 as compared with that of patient 9. This result shows that, like the *H*‐index, the *HI* method can give incorrect information about dose homogeneity. Fig. 5(b) indicates that, based on the *S*‐index, the dose homogeneity of the DVH is 17% better for patient 6 than for patient 9, which indicates that the *S*‐index uniquely provides quantitatively accurate information on dose homogeneity.

**Figure 6:**
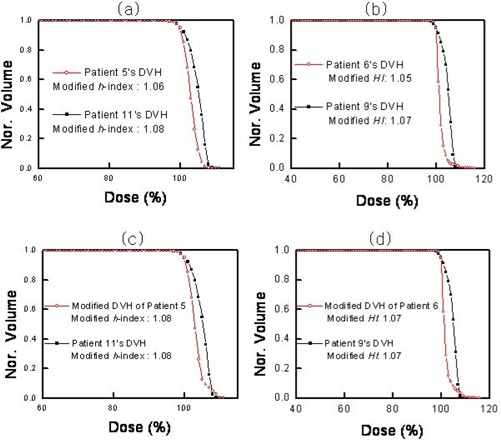
(a) Modified conventional homogeneity indices (*H*‐indices) for the two dose–volume histograms (DVHs) in Fig. 5(a). (b) Modified homogeneity index *HI* for the two DVHs in Fig. 6(a). (c) Modified *H*‐indices for the modified DVHs of patients 5 and 11. (d) Modified *HI* indices for the modified DVHs of patients 6 and 9. Nor.=normalized volume.

Although the *S*‐index is recommended for evaluating dose homogeneity, the accuracies of the conventional homogeneity indices can be improved by modifying their definitions. For instance, the *H*‐index can be made more robust by using $D_{5}$ instead of $D_{\max}$ (modified *H*‐index), and *HI* can be improved by using $D_{5}$ and $D_{95}$ instead of $D_{2}$ and $D_{98}$ (modified *HI*). Fig. 6(a,b) shows the modified *H*‐index and the modified *HI* for the DVHs shown in Figs. 4(a) and 5(a). Now, just as the *S*‐index does, the modified *H*‐index and the modified *HI* both appear to provide appropriate values for the dose homogeneity of the DVHs.

However, the modified indices still have problems. Fig. 6(c) shows two DVHs. One is the same as that shown in Fig. 6(a) for patient 11; the other is an artificially modified DVH for patient 5. The figure illustrates a slight change in the hot spots between the original and modified DVH for patient 5. In other words, the modified DVH of patient 5 is still more homogeneous than that of patient 11; however, the dose homogeneities are now the same based on the comparison using the modified *H*‐index. Moreover, Fig. 6(d) shows that the *HI* system also gives incorrect information. Although the modified DVH of patient 6 is more homogeneous than that of patient 9, the modified *HI* values incorrectly indicate that the dose homogeneities are the same.

We believe that any homogeneity index based on the doses at only a limited number of points of the DVH may provide incorrect information about dose homogeneity in the PTV. Moreover, another point should be considered when evaluating the dose homogeneity of a treatment plan: namely, the radiobiologic impact of dose inhomogeneity on the PTV.

The concept of the equivalent uniform dose (EUD) for tumors as proposed by Niemierko is one of the methods used to show the relationship between the dose homogeneity and radiobiologic effects.^(^ ^11^ ^,^ ^12^ ^)^ The EUD is defined as the biologically equivalent dose that, if given uniformly, would lead to the same reduction in the tumor volume as the actual dose that has an inhomogeneous distribution. Therefore, if the homogeneity index is properly constructed, it should be closely related to the EUD.

**Figure 7:**
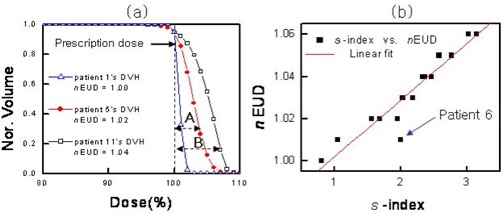
(a) Three dose–volume histograms (DVHs) with corresponding normalized equivalent uniform dose (*n*EUD) values. (b) Near‐linear relationship between the sigma index (*S*‐index) and *n*EUD. Nor.=normalized volume.

Fig. 7(a) shows the DVHs and corresponding normalized EUD (*n*EUD) values for three patients, where the *n*EUD is defined as the ratio of the EUD to the prescription dose. The figure shows that *n*EUD increases with the overdose‐induced inhomogeneity. For example, the *n*EUD of the DVH of patient 11 is larger than that of patient 5 because the overdose‐induced inhomogeneity is larger in patient 11 [labeled “B” in Fig. 7(a)] than in patient 5 [labeled “A” in Fig. 7(a)]. In general, the mean dose and the *n*EUD in the PTV will both increase with the overdose‐induced inhomogeneity in the DVH, which suggests a proportionality relationship between the *S*‐index and the *n*EUD.

Fig. 7(b) shows the high linearity of the fit between the *n*EUD data of 16 patients at our institution and their corresponding *S*‐indices. Note that the outlier for patient 6 in Fig. 7(b) is probably the result of the relatively long overdose tail in this patient's DVH [see Fig. 5(a)], which seems to increase the *S*‐index without a big change in *n*EUD value. Just as the same radiobiologic effect results if two *n*EUD values are the same, the equivalent value of the *S*‐index usually seems to provide the same radiobiologic effect. Here, we assumed that the two DVHs are normalized, so that 95% of the PTV receives the prescription dose. Although the *S*‐index closely reflects the *n*EUD value in general, two DVH curves can be seen to have the same standard deviation (that is, the same *S*‐index) and different *n*EUD values (different biologic effects) under certain conditions—as seen in the outlier for patient 6 in Fig. 7(b).

For simple evaluation of the dose homogeneity of a plan, the degrees of dose homogeneity of the DVHs have been categorized arbitrarily based on the *S*‐index as indicated in Table 2. The DVHs with *S*‐indices of less than 2 (meaning less than 2% standard dose deviation in the target volume) were classified as “outstanding” dose homogeneity. The *n*EUD values in this category typically show doses that differ by 1% from the prescription dose. The DVHs of the next grade (those for which the *S*‐index is greater than 2 but less than 2.5) were classified as “good.” The Radiation Therapy Oncology Group (RTOG) has provided a guideline for clinically acceptable DVHs.^(13)^ The no‐variation conditions recommended by RTOG are these:

- No more than 20% of any PTV will receive more than 110% of its prescribed dose.
- The prescription dose is the isodose that encompasses at least 95% of the PTV.
- No more than 1% of any PTV will receive less than 93% of its prescribed dose.

It turns out that the treatment plans classified “good” or “outstanding” based on the *S*‐index meet the criteria recommended by the RTOG for the no‐variation condition.

**Table 2:** Comparison of sigma indices (S‐indices) with mean normalized equivalent uniform dose (nEUD) values for various dose homogeneities

*S*‐index (*n*)	DVHs of *n*EUD	Average between $D_{p}$ and EUD	Percentage difference (PD) of homogeneity	Class
0≤S<2	5	1.01	PD<2%	Outstanding
2≤S<2.5	6	1.03	2%<PD<4%	Good
2.5≤S<2.5	5	1.05	4%<PD<6%	Fair
n≥3.5	0	‐	PD>2%	Poor

## IV. CONCLUSIONS

We have used statistical analyses of DVHs to propose a simple index for the objective assessment of the dose homogeneity of radiotherapy in the PTV. We believe that this new index is superior to conventional methods such as the *H*‐index and the *HI* because it provides complete information for the entire DVH curve in a treatment plan. The results presented here show that the *S*‐index provides better evaluation of the degree of dose homogeneity. It has been also shown that the *S*‐index is generally proportional to the *n*EUD, where *n*EUD is the quantity known to be related to the inhomogeneity‐induced radiobiologic impact to the tumor.

## ACKNOWLEDGMENT

This investigation was supported by a research grant from the National Cancer Center, Korea (No. 0610060).
